# Optimizing Postoperative Opioid Prescribing Through Quality-Based Reimbursement

**DOI:** 10.1001/jamanetworkopen.2019.11619

**Published:** 2019-09-18

**Authors:** Ryan Howard, Alexander Hallway, Jessica Santos-Parker, Joceline Vu, Jennifer Waljee, Chad M. Brummett, Michael Englesbe

**Affiliations:** 1 Michigan Opioid Prescribing and Engagement Network, Ann Arbor; 2 Department of Surgery, University of Michigan Health System, Ann Arbor; 3 University of Michigan Medical School, Ann Arbor; 4 Section of Plastic Surgery, Department of Surgery, University of Michigan Health System, Ann Arbor; 5 Department of Anesthesia, University of Michigan Health System, Ann Arbor; 6 Section of Transplant Surgery, Department of Surgery, University of Michigan Health System, Ann Arbor

## Abstract

This quality improvement study examines the use of a novel reimbursement incentive for surgeons to counsel patients on an opioid-sparing pathway for postoperative pain management.

## Introduction

Excessive postoperative opioid prescribing contributes to opioid dependence and morbidity.^1^ Encouragingly, a 2019 study^2^ found that an opioid-sparing postoperative recovery pathway was associated with minimal opioid use, low pain scores, and high patient satisfaction. Given the increasing rates of opioid dependence and mortality, this study examines a novel reimbursement incentive to mitigate excessive opioid prescribing after surgical operations.

## Methods

This quality improvement study was deemed exempt from regulation by the institutional review board of the University of Michigan because the exposure was not outside of normal clinical practice. This study follows the Standards for Quality Improvement Reporting Excellence (SQUIRE) reporting guideline.

In August 2018, the Michigan Opioid Prescribing and Engagement Network developed an opioid-sparing postoperative pathway for managing pain.^2,3^ Preoperatively, patients receive counseling about managing their pain without opioids. Postoperatively, patients receive prescriptions for acetaminophen and ibuprofen for all-day use for 1 week. In addition, they received a predetermined prescription for four to ten 5-mg oxycodone tablets intended for use in the first 1 to 2 days after the surgical operation.^4^

Simultaneously, Blue Cross Blue Shield of Michigan incentivized this opioid-sparing pathway by allowing the use of a modifier 22 for *unusual procedural service*. Modifier 22, as classified by *Current Procedural Terminology*, allows for additional reimbursement when the work required to provide a service is substantially greater than what is typically required. Surgeons could receive an additional 35% reimbursement to professional fees to recognize the additional care required for counseling patients on expectations for pain and recovery, use of nonopioid pain medications, and appropriate use and disposal of opioids. At the University of Michigan Health System, this was reflected in surgeon relative value units and payable to the surgeon’s department. Surgeons were required to attest in the operative report that the pathway was followed. Information regarding this new incentive was disseminated to surgeons through department meetings and email. Surgeons determined whether a patient was eligible for this pathway at their discretion. The primary outcome was the number of operations that used the opioid-sparing pathway, determined by documentation of counseling and by prescriptions for acetaminophen, ibuprofen, and the predetermined amount of 5-mg oxycodone tablets.

## Results

From August 1, 2018, to March 31, 2019, 1459 operations were performed by 51 surgeons across 6 procedures (Table), of which 1027 (70.4%) used the opioid-sparing postoperative pathway. Median (interquartile range) utilization of this pathway was 91.7% (51.6%-95.6%), ranging from 19.3% for laparoscopic cholecystectomy to 96.8% for robotic prostatectomy. Surgeons attested to using the pathway in 513 operative reports (50.0%) and received 35% additional reimbursement, which ranged from $389 to $753 per operation. An additional 514 operations (50.0%) used this pathway but did not have the attestation required for additional reimbursement. Attestation rate varied by procedure, from 23.9% for endoscopic sinus operations to 67.1% for thyroidectomy. The rate of surgeon attestation increased from 38.1% to 60.7% during 8 months (Figure).

**Table.** Use of an Opioid-Sparing Pathway From August 2018 to March 2019

Procedure	Operations, No.			Rate, %	
	Eligible	Used Pathway	Attested Pathway Use	Utilization	Attestation
Robotic prostatectomy	284	275	144	96.8	52.4
Endoscopic sinus operation	221	138	33	62.4	23.9
Laparoscopic					
Cholecystectomy	368	71	32	19.3	45.1
Inguinal hernia repair	126	120	41	95.2	34.2
Sleeve gastrectomy	161	146	77	90.7	52.7
Thyroidectomy	299	277	186	92.6	67.1

**Figure.**
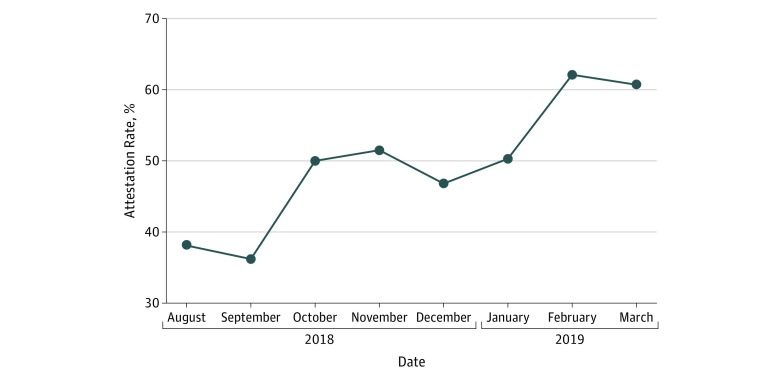
Change in Attestation Rate From August 2018 to March 2019

## Discussion

Although prescribing recommendations have been offered as a strategy to reduce excessive opioid prescribing, dissemination and uptake into routine clinical care have been slow.^5^ Physician incentives in the form of value-based or quality-based payments have been suggested as a novel strategy to immediately address opioid morbidity, primarily in the setting of addiction treatment.^6^ To our knowledge, this is the first example of financially incentivizing a preventive strategy. In 8 months after implementation, we observed rapid adoption, with utilization exceeding 90% in 4 of 6 procedures. Interestingly, clinical change outpaced the financial incentive component, as surgeons included the attestation required for additional reimbursement only 50% of the time.

This study has limitations. A limitation that may explain the low attestation rate is that data were collected from a single academic institution where surgeon salary is not directly affected by insurance reimbursement. Expansion of this program to nonacademic surgical practices may therefore generate an even larger impact. Currently, this incentive is available throughout Michigan.

In this quality improvement study, a quality-based payment program that incentivized surgeons to use an opioid-sparing recovery pathway was associated with rapid practice change. This may represent an effective strategy to immediately affect the opioid crisis by reducing excessive opioid prescribing after surgical operations.
